# Hepatic Gene Expression Changes in Rats Internally Exposed to Radioactive ^56^MnO_2_ Particles at Low Doses

**DOI:** 10.3390/cimb43020055

**Published:** 2021-07-22

**Authors:** Bakhyt Ruslanova, Zhaslan Abishev, Nailya Chaizhunussova, Dariya Shabdarbayeva, Sholpan Tokesheva, Gaukhar Amantayeva, Ynkar Kairkhanova, Valeriy Stepanenko, Masaharu Hoshi, Nariaki Fujimoto

**Affiliations:** 1 Department of Pathological Anatomy and Forensic Medicine, Semey Medical University, Semey 071400, Kazakhstan; baharuslanova@gmail.com (B.R.); zhaslan_love@mail.ru (Z.A.); dariya_kz67@mail.ru (D.S.); 2 Department of Public Health, Semey Medical University, Semey 071400, Kazakhstan; n.nailya@mail.ru (N.C.); sholpan.man.77@mail.ru (S.T.); gauhar2101@mail.ru (G.A.); 3 Department of Microbiology, Semey Medical University, Semey 071400, Kazakhstan; Inkar1357@mail.ru; 4 National Medical Research Center of Radiology, Ministry of Health of Russian Federation, 249031 Obninsk, Russia; valerifs@yahoo.com; 5 The Center for Peace, Hiroshima University, Hiroshima 7300053, Japan; mhoshi@hiroshima-u.ac.jp; 6 Research Institute for Radiation Biology and Medicine, Hiroshima University, Hiroshima 7340037, Japan

**Keywords:** residual radiation, internal radiation exposure, hepatic gene expression, radiation-sensitive genes, radiation-induced liver damages

## Abstract

We have studied the biological effects of the internal exposure to radioactive manganese-56 dioxide (^56^MnO_2_), the major radioisotope dust found in soil after atomic bomb explosions. Our previous study of blood chemistry indicated a possible adverse effect of ^56^MnO_2_ on the liver. In the present study, we further examined the effects on the liver by determining changes in hepatic gene expressions. Male Wistar rats were exposed to ^56^MnO_2_ particles (three groups with the whole-body doses of 41, 90, and 100 mGy), stable MnO_2_ particles, or external ^60^Co γ-rays (2 Gy), and were examined together with the non-treated control group on postexposure day 3 and day 61. No histopathological changes were observed in the liver. The mRNA expression of a p53-related gene, the cyclin-dependent kinase inhibitor 1A, increased in ^56^MnO_2_ as well as in γ-ray irradiated groups on postexposure day 3 and day 61. The expression of a stress-responsive gene, nuclear factor κB, was also increased by ^56^MnO_2_ and γ-rays on postexposure day 3. However, the expression of cytokine genes (interleukin-6 or chemokine ligand 2) or fibrosis-related TGF-β/Smad genes (*Tgfb1*, *Smad3*, or *Smad4*) was not altered by the exposure. Our data demonstrated that the internal exposure to ^56^MnO_2_ particles at less than 0.1 Gy significantly affected the short-term gene expressions in the liver in a similar manner with 2 Gy of external γ-irradiation. These changes may be adaptive responses because no changes occurred in cytokine or TGF-β/Smad gene expressions.

## 1. Introduction

At the atomic bombing of Hiroshima and Nagasaki, Japan, initial radiation directly from the explosions caused major biological impacts. However, people who entered these cities soon after the detonations and who did not receive any direct radiation were reported to suffer from acute radiation syndrome, such as nausea, vomiting, and diarrhea [1]. These people might have inhaled residual radioactive dust and may have been exposed to radiation. Main radioisotopes produced in soil by the neutron from the atomic bomb explosions included ^24^Na, ^42^K, and ^56^Mn [2]. As ^56^Mn (a β/γ emitter with a half-life of 2.58 h) exists as insoluble ^56^MnO_2_ particles in the soil, it would be the most critical source of residual radiation.

Then, we conducted animal studies examining the biological effects of internal exposure to ^56^MnO_2_ particles [3]. Male Wistar rats were exposed to ^56^MnO_2_ produced by neutron beam from a reactor. The accumulated radiation doses were high in the gastrointestinal tract, followed by in the skin and the lung, with whole-body doses ranging from 41–100 mGy [3]. The blood chemistry showed that ^56^MnO_2_ exposure decreased serum alanine aminotransferase (ALT) on day 3 but increased on day 61 postexposure, suggesting some adverse effects on the liver [4]. Therefore, in the present study, we focused on the effects of internal exposure of ^56^MnO_2_ particles on the liver.

The effects of external radiation on hepatic gene expressions have been investigated extensively in earlier studies [5,6,7]. Irradiation of the liver at high doses (>20–30 Gy) may induce progressive liver fibrosis and cirrhosis [8,9,10]. Several studies showed that expressions of fibrosis-related genes such as *Tgfb1*, *Smad3*, and *Smad4* are elevated after irradiation [11,12,13]. At the lower doses (6–8 Gy), the irradiation induces moderate histopathological changes, including Kupffer cell activation and occasional fatty change, which accompany the elevated expression of cytokines as well as their activator, nuclear factor κB (NFκB) [14,15]. The cDNA microarray analysis has become an essential tool for identifying genes involved in tissue damage caused by irradiation and for understanding the molecular mechanisms of radiation sensitivity or resistance [16]. This method is useful to the study of the molecular pathways of physiological or adaptive responses to radiation in the liver [6]. A microarray study in the mouse liver showed that even a γ-irradiation of 1Gy changes the expression of genes such as cyclin-dependent kinase inhibitor 1A (*Cdkn1a*) [17]. The expressions of these genes are radiation-inducible and are useful markers for radiation effects. We examined these gene expressions to evaluate the short-term biological responses of internal exposure to ^56^MnO_2_ particles in the liver. The male Wistar rats were exposed to ^56^MnO_2_ particles with three different specific radio-activities and were examined on postexposure day 3 and day 61 to quantify the hepatic mRNA expression levels.

## 2. Materials and Methods

### 2.1. Animals

The animal experiment design was described previously [4]. Specific-pathogen-free male Wistar rats (10 weeks old) were purchased from Kazakh Scientific Center of Quarantine and Zoonotic Diseases, Almaty, Kazakhstan. They were maintained with free access to a basal diet and tap water. The animal facility was of the conventional type with a room temperature between 19 °C and 22 °C, relative humidity of 30–70%, and a 12 h light cycle. The rats were randomly divided into 6 groups: Mn56x1 group (*n* = 17), Mn56x2 group (*n* = 17), Mn56x3 group (*n* = 17), Co60 group (*n* = 14), cold Mn group (*n* = 14), and control group (*n* = 14). Mn56x1, Mn56x2, and Mn56x3 groups were exposed to 3 different activities of ^56^MnO_2_ powder (100 mg) of 2.7 × 10^8^, 5.5 × 10^8^, and 8 × 10^8^ Bq, respectively. The Co60 group received 2 Gy of external ^60^Co γ-ray whole-body irradiation. The cold Mn group was exposed to nonradioactive MnO_2_ powder (100 mg). All groups of rats including the non-treated control were taken to the ^56^MnO_2_ exposure facility to maintain the same experimental condition.

On postexposure day 3 and day 61, 7 rats were necropsied from each group. The rats were anesthetized with isoflurane gas (Fujifilm Wako Pure Chemical Co., Tokyo, Japan), and then euthanized by removing whole blood from an abdominal artery. The liver was dissected, and pieces of the left lobe were stored in RNA Save solution (Biological Industries Ltd., Beit Alfa, Israel) for RNA extraction. The rest of the liver was fixed in 10% formalin for pathological analysis.

Ethics Committee Approval (document #3-30.11.2018) was obtained from the Animal Experiment Ethics Committee of Semey Medical University, Semey Kazakhstan before commencing the study.

### 2.2. Irradiation

Details of irradiation using ^56^MnO_2_ powder and the dose estimation were described previously [18]. MnO_2_ powder (Rare Metallic Co., Tokyo, Japan) with particle diameters ranging from 1–19 µm (average diameter: 6 µm) was radioactivated by neutron beam in the Baikal-1 nuclear reactor at the National Nuclear Center, Kurchatov, Kazakhstan. The obtained ^56^MnO_2_ powder was air-pressure sprayed into sealed exposure boxes containing 8 or 9 rats per box (length 400 mm × width 200 mm × height 250 mm). After one hour, the animals were transferred to new cages to end the exposure. Three rats from each Mn56 group were necropsied for dosimetry. Absorbed doses of internal radiation were estimated based on measured radioactivity in each organ. The γ-ray irradiation was performed with a ^60^Co radiotherapy machine, Teragam K-2 unit (UJP Praha, Praha-Zbraslav, Czech Republic) at 2 Gy (1.0 Gy/min).

### 2.3. Pathology

Formalin-fixed tissues were embedded in paraffin. Sections of 4 µm thickness were prepared and stained with HE.

### 2.4. Measurement of mRNA Levels by Quantitative RT-PCR

Total RNA was prepared using Isogen II (Nippon Gene Co., Tokyo, Japan) from pieces of hepatic tissues stored in RNA Save solution. The cDNA was synthesized by incubating 3 µg total RNA with 100 U of ReverTra Ace reverse transcriptase (Toyobo Co., Osaka, Japan) with a mixture of 20 pmol random hexamers pdN6 and 5 pmol oligo-dT(15) primers (Takara Bio Inc., Kusatsu, Japan). A quantitative PCR instrument, StepOnePlus (Applied Biosystems/Life Technologies Co., Carlsbad, CA, USA), was employed to measure cDNA with a Thunderbird Next Sybr qPCR reagent (Toyobo Co.). The DNA sequences of the PCR fragments for target genes were confirmed by Fasmac Co., Ltd. (Atsugi, Kanagawa, Japan). PCR conditions were 20 s initial denaturation followed by 40 cycles of 5 s at 95 °C and 35 s at 60 °C. The measured mRNA levels were normalized by the levels of β-actin mRNA [19,20]. Table 1 shows the specific primer sets for the genes.

### 2.5. Statistical Analysis

All values are expressed as mean ± standard error of the mean. Williams’ test was performed to compare the mRNA levels between the three different dose groups and the cold Mn or the control group. Dunnett’s test was applied to compare the mRNA levels between the Co60 group and the cold Mn or the control group. Dunnett’s test was also used for body and liver weight comparisons between groups.

## 3. Results

### 3.1. Radiation Doses

The radiation doses of internal irradiation from ^56^MnO_2_ in each organ were previously reported [3]. The whole body doses of internal irradiation were 41 ± 8 mGy (Mn56x1 group), 91 ± 30 mGy (Mn56x2 group), and 100 ± 10 mGy (Mn56x3 group), while the absorbed doses of the liver were 1.5 ± 0.3, 4.5 ± 1.2, and 7.1 ± 1.6 mGy, respectively. The external irradiation dose of ^60^Co-γ exposure was 2.0 ± 0.08 Gy.

### 3.2. Body and Liver Weights

Table 2 shows the body and liver weights on postexposure day 3 and day 61. On day 61, both absolute and relative liver weights were significantly increased only in the Mn56x1 group without indicating any radiation dose-responsive changes. There were no other differences in body or liver weight among the groups.

### 3.3. Histology of the Liver

**Figure 1:**
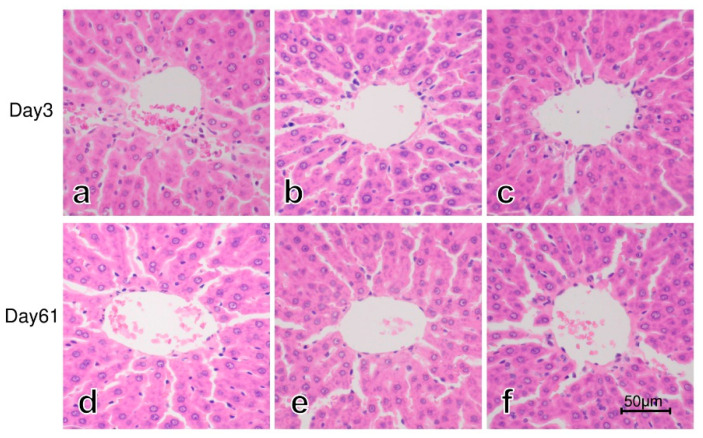
Liver of rats on day 3 (**a**–**c**) and day 61 (**d**–**f**) after the exposure to ^56^MnO_2_ particles or ^60^Co-γ rays. No significant histological changes were noted in Mn56x3 (**a**,**d**), Co60 (**b**,**e**), and the control (**d**,**f**) groups. HE staining, original magnification 400×. A bar indicates 50 µm.

Figure 1 shows representative hematoxylin and eosin (HE) staining of the liver on postexposure day 3 and day 61 in Mn56x3, Co60, and the control group. No significant histopathological changes were found in the liver of rats exposed to ^56^MnO_2_ or ^60^Co-γ rays.

### 3.4. Effects on mRNA Expression Levels of Cdkn1a, BCL2 Associated X (Bax), and Growth-arrest-and-DNA-damage-inducible 45 (Gadd45)

**Figure 2:**
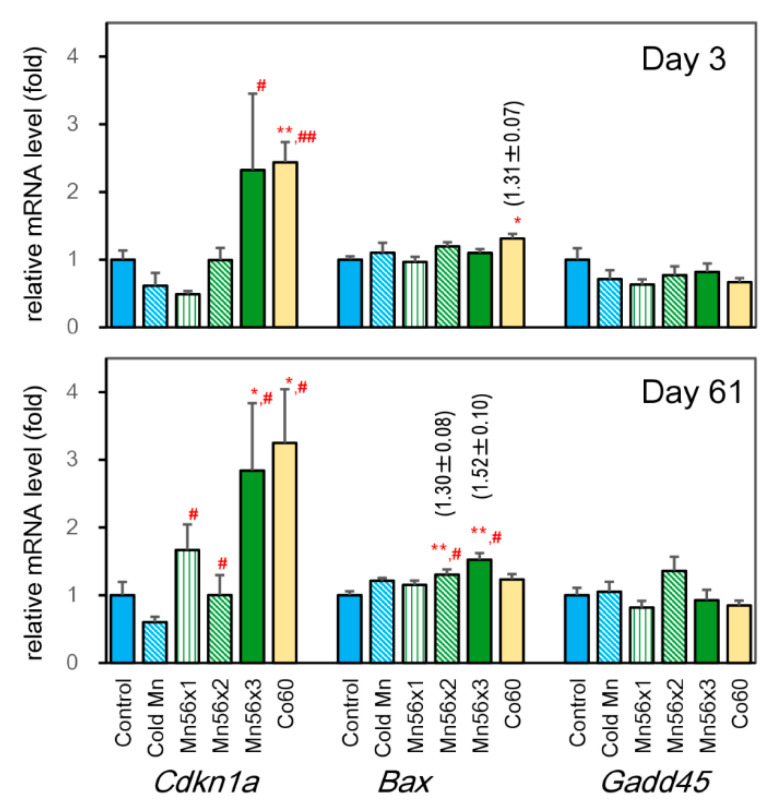
Relative mRNA expression levels of *Cdkn1a*, *Bax*, and *Gadd45* genes in the liver of rats on day 3 (top) and day 61 (bottom) after the exposure. Control (solid blue): untreated group; Cold Mn (diagonal blue stripes): exposed to stable MnO_2_ particles; Mn56x1 (green stripes), Mn56x2 (diagonal green stripes), and Mn56x3 (solid green): exposed to ^56^MnO_2_ particles at the activity of 2.6 × 10^8^, 5.5 × 10^8^, and 8 × 10^8^ Bq, respectively; Co60 (solid yellow): exposed to ^60^Co γ-rays (2 Gy). * *p* < 0.05 and ** *p* < 0.01 vs. control; # *p* < 0.05 and ## *p* < 0.01 vs. Cold Mn.

Figure 2 shows the expression levels of *Cdkn1a*, *Bax*, and *Gadd45* genes. On day 3 postexposure, *Cdkn1a* mRNA levels were significantly increased in both Mn56x3 and Co60 groups over the cold Mn group, while the increase was significant only in the Co60 group in comparison with the control group. The *Bax* expression was elevated by 29% in the Co60 group on day 3 postexposure, while it increased in Mn56x2 and Mn56x3 groups on day 61. There were no significant changes in *Gadd45* expressions among groups.

### 3.5. Effects on mRNA Levels of NFκB (Nfkb1), Interleukin (Il6), Chemokine Ligand 2 (Ccl2), TGF-β1 (Tgfb1), Smad3, and Smad4

**Figure 3:**
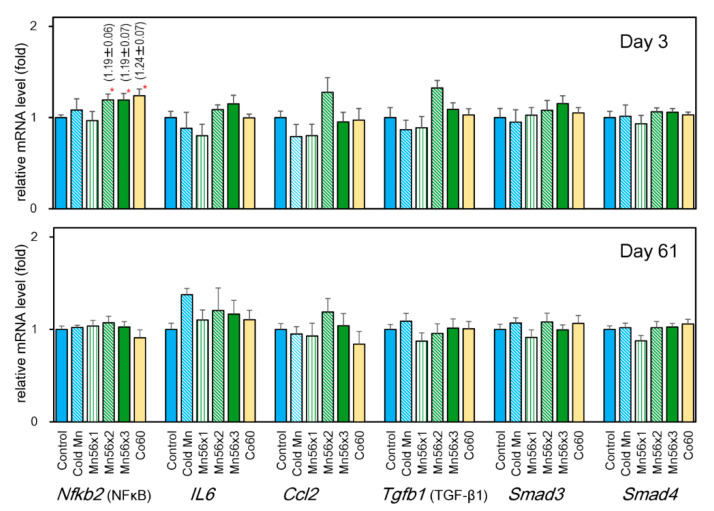
Relative mRNA expression levels of *Nfkb1*, *Il6*, *Ccl2*, *Tgfb1*, *Smad3*, and *Smad4* genes in the liver of rats on day 3 (top) and day 61 (bottom) after the exposure. Control (solid blue): untreated group; Cold Mn (diagonal blue stripes): exposed to stable MnO_2_ particles; Mn56x1 (green stripes), Mn56x2 (diagonal green stripes), and Mn56x3 (solid green): exposed to ^56^MnO_2_ particles at the activity of 2.6 × 10^8^, 5.5 × 10^8^, and 8 × 10^8^ Bq, respectively; Co60 (solid yellow): exposed to ^60^Co γ-rays (2 Gy) * *p* < 0.05 vs. control.

Figure 3 summarizes the mRNA expression of *Nfkb1*, *Il6*, *Ccl2*, *Tgfb1*, *Smad3*, and *Smad4*. On postexposure day 3, *Nfkb1* mRNA expression significantly increased in the Mn56x2, Mn56x3, and Co60 groups either compared with cold Mn or the control group, while these changes disappeared on day 61 postexposure. There were no significant changes in the expression of *Il6*, *Ccl2*, *Tgfb1*, *Smad3*, or *Smad4* among groups on either day examined.

## 4. Discussion

The present study investigated the effect of ^56^MnO_2_ exposure on hepatic gene expression. The quantitative RT-PCR data showed that internal exposure to ^56^MnO_2_ particles significantly enhanced the mRNA expression of the genes including Cdkn1a, Bax, andNfkb1, which were comparable with the effects of external γ-irradiation at 2 Gy. These data suggested that internal exposure of ^56^MnO_2_ could lead to significant short-term biological responses in the liver, even at less than 0.1 Gy of whole-body doses of internal irradiation.

For understanding the radiation effects among the atomic bomb survivors, evaluation of the effects of residual radiation is crucial. Neutron-activated radioisotopes were produced in the soil by the atomic bomb. People might have been affected by exposure to these radioactive materials. Those who came to Hiroshima soon after the atomic bombing without exposure to the initial radiation were reported to suffer from acute radiation syndrome [1]. The possible radioisotopes produced in soil by neutron from the atomic bomb included ^24^Na, ^42^K, and ^56^Mn [2]. Since ^56^Mn exists as insoluble MnO_2_ particles in the soil, the effects of this compound were investigated in rats. In a previous study, to assess possible systemic responses to this radioactive powder in rats, basic biological parameters, including the blood chemistry, were determined [4]. Only ALT significantly altered after exposure to ^56^MnO_2_ particles out of red and white blood cell counts, calcium, phosphorus, potassium, sodium, ALT, aspartate amino-transferase, amylase, creatinine, urea, protein, albumin, triglycerides, high density lipoprotein, total cholesterol, and glucose levels. Since an increase in ALT indicates possible adverse effects on the liver, the present study further examined the liver status by measuring the gene expressions related to hepatic damages.

In both human and animal models, it is well known that high doses of liver irradiation (>20–30 Gy) causes irreversible hepatic damage, including fibrosis, a major dose-limiting factor in radiotherapy [21]. When the rodent liver was irradiated at the above doses, it develops initial hepatocellular lesions, such as sinus congestion and fatty liver, followed by hepatic fibrosis 8 weeks or later [8]. Recent studies suggest the involvement of the TGF-β/Smad pathways in the development of liver fibrosis [11,13]. In the present study, as the histological examination indicated no fibroblastic changes in the liver, there were indeed no significant changes in *Tgfb1*, *Smad3*, or *Smad4* expressions by ^56^MnO_2_ or ^60^Co-γ exposure. Irradiation at lower doses (6–8 Gy) also causes hepatocellular damages that could be recovered [8]. At these doses, the cytokine expressions were induced due to the oxidative stress caused by irradiation [22,23,24]. In rats exposed to whole-body γ-irradiation at 6–7 Gy, the expression of cytokines including IL-6, TNF-α, and CCL2 (also known as monocyte chemoattractant protein 1, MCP-1), was increased, probably through the NFκB signaling pathway [14,15]. Although no increases were noted in the *Il-6* or *Ccl2* expressions in the present work, the Nfkb1 mRNA level was significantly induced by 2 Gy of ^60^Co-γ exposure, suggesting high sensitivity of the transcriptional regulation of NFκB to radiation. A cell culture study revealed that transcriptional activity of NFκB was indeed upregulated by as low as 1 Gy of X-rays [25]. Interestingly, the exposure to ^56^MnO_2_ particles also upregulated the NFκB expression showing the significant biological impact on the liver.

Microarrays have been applied to investigate the expression profiles in the irradiated liver to understand the molecular process responsible for radiosensitivity [5,6,7,26,27]. This method is advantageous to identify the effects of irradiation at low doses where the pathological effects are not evident. One study examined the gene expression profile in the liver of mice irradiated with 1 Gy of γ rays and reported quantitative changes in mRNA expression of 126 genes [17]. This study also showed that *Cdkn1A* was persistently upregulated during 10 weeks after irradiation. Our results in rats showed a similar long-lasting induction in *Cdkn1A* expression, which was significantly upregulated both on day 3 and day 61 after ^60^Co-γ irradiation. The increased expression of *Cdkn1A* was also evident in the 56Mnx3 group, showing the significant effects of internal exposure of ^56^MnO_2_. The expression of *Cdkn1A* is regulated by the transformation-related protein 53 (p53) [28]. In radiation-sensitive cells such as leukocytes and leukemia cell lines, the downstream genes of p53, *Bax*, and *Gadd45*, are significantly upregulated on irradiation [29,30,31]. However, the microarray analysis found that they were not altered in the mouse liver, indicating the liver tissue-specific regulation of the p53-related genes to radiation [17]. Our results in rat liver were consistent with these results, showing a limited increase in the Bax expression and no induction in *Gadd45* to radiation exposure.

## 5. Conclusions

To understand the biological effects of residual radiation after the atomic bomb explosion, the hepatic gene expression in the rats exposed to ^56^MnO_2_, one of the major radioactive compounds produced in soil by the atomic bomb, was investigated. The short-term upregulation of *Cdkn1A*, *Bax*, and *Nfkb1* gene expressions was found by the exposure to both ^60^Co-γ rays and ^56^MnO_2_ particles on day 3 postexposure, while *Cdkn1A* and *Bax* mRNA levels were still high on postexposure day 61. This study suggests that internal exposure to ^56^MnO_2_ particles, even at whole-body internal doses of less than 0.1 Gy, significantly impacts hepatic gene expressions, comparable to 2 Gy of external irradiation.

## Figures and Tables

**Table 1:** Q-PCR primers.

Gene	GenBank Accession Number	Q-PCR Primer Sequences (5′ → 3′)	
		Forward	Reverse
*Cdkn1a*	NM_080782	TGTCCGACCTGTTCCACACA	CGTCTCAGTGGCGAAGTCAA
*Bax*	NM_017059	TGTGGATACAGACTCCCCCC	TGATCAGCTCGGGCACTTTA
*Gadd45*	L32591	GAGTCAGCGCACCATAACTGTC	AATGAGGGTGAAATGGATCTGC
*Nfkb1(NFκB)*	LC369719	CCTGTCTGAAGCCCTGCTACA	CACACCCTGGTTCAGAAGCTG
*Il6*	NM_012589	TCACAGAGGATACCACCCACAA	TCTGACAGTGCATCATCGCTG
*Ccl2*	NM_031530	AAGCCAGATCTCTCTTCCTCCA	CAGCAACTGTGAACAACAGGC
*Tgfb1(TGFβ1)*	AY550025	GCTGAACCAAGGAGACGGAAT	GAAGGGTCGGTTCATGTCATG
*Smad3*	NM_013095	AGAACGTGAACACCAAGTGCAT	CCCGTAACTCATGGTGGCTG
*Smad4*	AB010954	ACGGAAGGACATTCGATTCAA	GACTTGTGGAAGCCACAGGAA

**Table 2:** Body and liver weights in rats exposed to ^56^MnO_2_, ^60^Co γ-rays, and cold MnO_2_.

Groups ^1^		Body Weight (g)	Liver (g)	Liver (Relative) (g/kg bw)
Day3	Control	248 ± 16	6.80 ± 0.40	28 ± 1.2
	Cold Mn	235 ± 14	7.08 ± 0.41	30 ± 0.4
	Mn56x1	235 ± 11	6.68 ± 0.40	28 ± 1.0
	Mn56x2	245 ± 16	6.86 ± 0.39	28 ± 0.8
	Mn56x3	237 ± 12	6.94 ± 0.37	29 ± 0.5
	Co60	234 ± 14	6.71 ± 0.34	29 ± 1.5
Day61	Control	330 ± 17	8.73 ± 0.54	26 ± 0.7
	Cold Mn	337 ± 19	10.8 ± 0.51	32 ± 1.0
	Mn56x1	371 ± 21	13.2 ± 0.71 **	36 ± 1.4 **
	Mn56x2	337 ± 17	8.94 ± 0.54	27 ± 1.0
	Mn56x3	353 ± 17	9.51 ± 0.40	27 ± 0.4
	Co60	328 ± 23	8.67 ± 0.70	26 ± 0.8

^1^ Control: untreated group; Cold Mn: exposed to stable MnO_2_ particles; Mn56x1, Mn56x2, and Mn56x3: exposed to ^56^MnO_2_ particles at the activity of 2.6 × 10^8^, 5.5 × 10^8^, and 8 × 10^8^ Bq, respectively; Co60: exposed to ^60^Co γ-rays (2 Gy). Each value shows mean ± SEM (*n* = 6 or 7, each group). ** indicates significant difference from the control at *p* < 0.01.
